# Measuring ancient technological complexity and its cognitive implications using Petri nets

**DOI:** 10.1038/s41598-023-42078-1

**Published:** 2023-09-22

**Authors:** Sebastian Fajardo, Paul R. B. Kozowyk, Geeske H. J. Langejans

**Affiliations:** 1 Department of Materials Science and Engineering, Delft University of Technology, 2628 CD Delft, Zuid-Holland The Netherlands; 2 Palaeo-Research Institute, University of Johannesburg, Johannesburg, 2092 Gauteng South Africa

**Keywords:** Archaeology, Computer science, Applied mathematics, Computational models

## Abstract

We implement a method from computer sciences to address a challenge in Paleolithic archaeology: how to infer cognition differences from material culture. Archaeological material culture is linked to cognition, and more complex ancient technologies are assumed to have required complex cognition. We present an application of Petri net analysis to compare Neanderthal tar production technologies and tie the results to cognitive requirements. We applied three complexity metrics, each relying on their own unique definitions of complexity, to the modeled production processes. Based on the results, we propose that Neanderthal technical cognition may have been analogous to that of contemporary modern humans. This method also enables us to distinguish the high-order cognitive functions combining traits like planning, inhibitory control, and learning that were likely required by different ancient technological processes. The Petri net approach can contribute to our understanding of technology and cognitive evolution as it can be used on different materials and technologies, across time and species.

## Introduction

Human origins and the evolution of cognition are intricately tied to the use of technology^1–6^. The development of complex technologies over the last 3.3 million years provides a mirror to the cognitive developments that underpin behavioral changes. Generally, the processes of production and use of archaeological objects are first (modeled and/or experimentally) reconstructed and then interpreted using concepts such as cognitive load, learning, reflectiveness, working memory, extended thought, and action sequences^7–10^. Oversimplifying one of the main hypotheses, it could be said that a more complex mind can give rise to more complex technologies and thus that we can reverse engineer cognition from technology and material culture. However, the link between the complexity of technologies and cognition remains qualitative, restricting systematic comparisons of different technological processes and their cognitive requirements.

Tar production, an example of complex technology, often features in discussions about Neanderthal and modern human technological and cognitive capabilities^1,11–13^. However, the exact complexity of birch tar technology is debated, as there are multiple ways to make tar without fireproof containers^14–16^. Recent experiments show that birch tar can be produced with simple methods^15^. However, none of the reconstructed methods have been systematically studied for their complexity with definitions for what is considered simple or complex that can contribute to the current technology and cognition debates. The simplest method, does require fewer materials and the production process consists of fewer unique steps than other techniques, but the implications of these criteria on complexity/cognition are unspecified. In this paper we take a step back in the debate and we explore a method to overcome these two problems of a) measuring technological complexity, and b) linking technology to cognition. We use Petri net modeling^17^ to compare the complexity of Neanderthal birch tar production methods in terms of the cognitive requirements of each method. This analysis is based on the examination of the events and conditions involved in the processes and the relationships between them. We utilize Petri nets as a method for modeling and quantifying system complexity for ancient tar production methods. By doing so, we establish a proxy for measuring the cognitive demands associated with these methods and other ancient technological processes.

The measurement and comparison of different ancient technological processes is often challenging due to the uniqueness of interrelations between cognitive processes and production events, and the sparse nature of the archaeological record. Various measures have been used in the past, such as counting techno-units in tool kits, steps in behavioral sequences, procedural units in the production of a specific tool, number of and/or decisions, and distance between a need and the satisfaction of that need^4,18–22^. These measurements are generally unique for specific tools and materials. In this paper we use Petri nets as a tool for expanding measurements of technological processes. Petri nets can model and study the causality and execution of events, including sequential, concurrent, and parallel execution^17^. This allows the identification of differences in the way information is processed to obtain or use a product. Additionally, we argue that with Petri nets, different definitions for complexity can be applied and compared for the same production process, exposing related behavioral and cognitive implications. Our approach moves away from focusing solely on verifying one particular dimension (definition) of technological complexity. Instead, we consider the differences between production methods in light of the type of solution a method represents.

The skilled technical cognition model implies that technologies, such as birch tar production, demand the retrieval and integration of a wide range of cognitive structures. Active attention is required to combine these structures during tar production^23^, and causal reasoning is required to infer forces that are not directly perceived^24,25^. Similarly, previous studies suggest that the production of Paleolithic or Stone Age adhesives requires the maker to be able to: a) accessing multiple pieces of information at the same time while executing the process; b) avoid errors and correct problems throughout the production process; c) understand and abstract information about the materials, product templates, and the process itself before starting to make adhesives^3,23,26^. We measured these requirements using Petri nets and discuss the implications of the results in light of the technical cognition models. Our aim is to gain a deeper understanding of the cognitive implications of current knowledge about the tar technologies employed in the Paleolithic.

Processing information at the same time (a) is a fundamental requirement in technological production. Production systems have different demands regarding the amount of information and the timing of information processing to execute the tasks and obtain the final product. The level of information processing needed at a given time is influenced by the number of interactions among elements involved in the tasks. Higher levels of interaction often imply a larger volume of information that needs to be processed simultaneously^27,28^. Moreover, such instances in a process involve the use of cognitive functions to focus attention^29^ and to reach the end goal of a task in the face of interference-rich contexts^29,30^. This becomes particularly relevant when conducting a technological process in an environment without permanent craft specialization.

Multiple permutations of events increase the likelihood of errors (b) and complexity. In a production process, different permutations create more possible paths to obtain the final product and increase the probability of deadlocks, wasting time and resources^31^. Complexity is also increased with more choices, because individuals may be unaware of all available choices, or what the best choices are to obtain the desired product. For these reasons, a sequential production process is also easier to execute than one with several paths to reach the end of the workflow^32^. Implementing strategic planning^33^ and inhibitory control^34^ may reduce the likelihood of errors produced by different permutations and choices and help to solve complex problems.

Process structures with more elements and relations are harder to understand (c) because more information needs to be processed. Since information presented in a simple arrangement is easier to understand than if the same information is presented in an elaborate structure, both the amount of information and the types of structures affect the structural complexity of a process. This in turn affects process understandability, which provides an indication of how much information is embedded in the process^35^. Previous studies have identified that understanding how to produce a technology was an important aspect in the acquisition, transmission, and production of Paleolithic technologies (e.g.^9,36^; but see also^37,38^).

The complexity of the production of prehistoric artifacts can be measured using the requirements introduced above in combination with computational models^39^. To do this, we used Petri nets to model Paleolithic tar production processes. Petri nets are a modeling language with underlying mathematical semantics^17,40^. These nets are used to study systems that may show concurrent agents or events and components that operate independently with occasional resource sharing or synchronization. We used workflow nets^41^, a class of Petri net, to model and measure the complexity of resulting models using three pre-existing metrics: (a) the density metric that takes into account the interconnectedness between events and resources^42^, and can be related to requirements of simultaneous information processing; (b) the extended cyclomatic metric^43^, that concerns the likelihood of errors throughout the process, and the potential need for planning and inhibition control; and c) the structuredness metric, which relates to the effort to understand abstract information about the materials, product templates and the process itself, and thus to learning^43^.

We model and measure three experimental techniques of birch bark tar production known from the literature: *condensation*^15^, *pit roll*, and *raised structure*^18^. Currently, the *condensation* method is interpreted to be the simplest of the three, and the *raised structure* the most complex. These methods cover the widest range of potential tar making techniques and represent our current knowledge about aceramic tar production, both in terms of yield, time invested, number of production steps, and materials required. Our focus lies in assessing the structure and state space of each tar production process. We are not primarily concerned with comparing mental models of tar makers. The Petri net models of these production processes and the results of the complexity metrics allow us to present a multidimensional comparison of the complexity of an ancient technology. With the metrics, we also aim to show that simultaneous information processing, inhibitory control, and planning are all cognitive requirements involved in the aceramic production of tar. This approach enhances our understanding of technological processes through modeling. It reveals often overlooked attributes like concurrency, thereby enriching technological analyses in archaeology.

## Methods

We modeled the tar production processes based on existing literature, observations of experimental executions, and knowledge from experts in Paleolithic tar production. In the *condensation* method a piece of bark is burnt next to a cobble, tar fumes condense against the rock, and tar can be periodically scraped off^15^. This process is repeated again until a small lump of tar is collected. For the *pit roll* method, a roll of bark is fit into a small hole in the ground and embers are used to burn the bark from the top. As tar is formed inside the roll, it drips into a container at the bottom of the hole^18^. In the *raised structure*, a birch bark vessel is placed in a hole in the ground. A screen of small twigs is placed over the hole, and a large roll of birch bark is placed over the screen. This is covered with muddy soil to form a dome or oven-like structure. A fire is lit around the structure for several hours. Any holes in the dome are fixed before or during heating as needed. The tar that forms drips through the twigs and into the underground container^18^.

We recreated the tar production processes in field experiments. Non-participant observation was implemented to record the process of each experiment^44^. S.F. observed and recorded the experiments and the actions of P.R.B.K and G.H.J.L with a video camera, and time stamped all events during the coding phase. After the experiments, P.R.B.K was asked to describe the workflow of the experiments from the executant’s perspective.

These different sources were integrated to produce comprehensive models of the actions, events, conditions, and sequences that could occur during the workflow. These models may be used to generate system description files that are essential for analyzing the cognitive complexity of actual task-solving process descriptions translated into log files^45^.

### Petri nets

**Figure 1:**
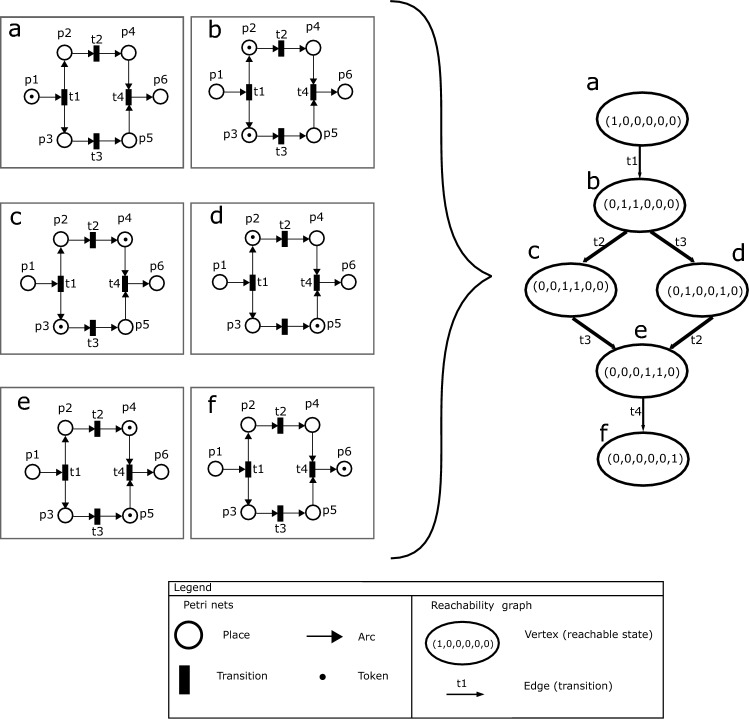
A workflow net graphic representation and its reachability graph to illustrate dynamics of Petri nets. The nets on the left show the states of a Petri net that consists of six places labeled p1 to p6, four transitions labeled t1 to t4, and ten arcs connecting them. All arcs and transitions have the same function that returns 1, meaning that any transition can fire as long as its input places are marked with tokens. The graph on the right of the curly bracket is the reachability graph of the Petri net. Ovals represent states with token counts indicated inside, from left to right: p1 to p6. Arrows symbolize transitions and are labeled with corresponding occurrences. In the initial reachable state of the Petri net (**a**), only place p1 has one token, and all other places are empty. When t1 fires, the marking of the Petri net changes and places p2 and p3 have one token each (**b**). Then transitions t2 and t3 can occur in any order, including in parallel, to produce tokens in p4 and p5, respectively, as shown in (**c**) and (**d**). After places p4 and p5 are marked with one token each (**e**), then transition t4 may occur and produce a token in p6 to reach the final reachable state of the Petri net (**f**).

Petri nets have three basic elements: places, transitions, and arcs (e.g. Fig. 1)^17,40^. *Places* represent states, conditions, or resources that need to be met or available before an event can be carried out. They also represent the result of an event, i.e., a condition or resource. Places cannot be directly connected with one another. Places are depicted as circles in the Petri net graphic representation. *Transitions* represent events. A transition may occur when all the required conditions or resources of its input places are available. Two transitions cannot be directly connected. The firing of a transition is instantaneous and the choice of which transition to fire when several transitions may occur concurrently is random. Transitions are shown as black rectangles in the graphic representation. *Arcs* represent the flow of the system and connect places and transitions. They indicate which places (i.e., conditions or resources) are required for enabling a transition and to which places the resources or conditions resulting from a transition are directed. In the graphic representation arcs are shown as arrows. By combining these three elements, Petri nets provide a formal, graphical, and executable model of the system.

In a Petri net the availability of resources or the fulfillment of conditions is graphically represented using black dots or numbers called tokens. Tokens are placed inside places. The distribution of tokens in different places at any given moment represents the current state of the system. Taken together, the states of the system provides insight into the workings of the system over time. These states are also called markings in Petri nets.

In workflow nets, arcs have a weight of one because places correspond to conditions that can be validated as true or false. Processes modeled as workflow nets start with a token marking one unique input place and should always be able to end with a token in a different unique output place, with all the other places being empty. In workflow nets, transitions can always occur by following the appropriate route in the workflow and they do not create infinite loops^41,43^.

### Modeling approach

The tar production models relied on a set of assumptions. First, we focused on the intrinsic variability of tar production processes, rather than the way environmental settings determine the availability of resources. Therefore, we assumed that cultural, social, and environmental restrictions related to resource availability did not play a role in the workings of the processes. Second, we considered that resources, tools, and time required for activities were available and did not represent behavioral constraints. Third, models were developed as action oriented, and people executing actions were excluded from the models.

For the models presented here, we defined the atomic units as events that changed the location or modified the physical properties of resources. For example, in the *pit roll* and *raised structure* methods, a roll is created using a piece of bark. Making this roll represents an event that modifies the shape, a qualitative physical property, of the birch bark. In the tar production models, places represented either the presence/absence of a material, or whether an action occurred. The models ran a single process instance. This enabled the use of pre-existent complexity metrics and controlled the effects of parameters such as the required amount of tar. Assuming infinite resources and time, any of the tar production techniques can be repeated as many times as needed to obtain a specific amount of tar, but these repetitions do not change the workings of each process. For example, for the *condensation* method, we modeled the events from when one piece of bark was burned, until the tar produced by that piece of bark was stored. However, in practice, this method involved burning several pieces of bark and extracting and storing tar repeatedly in the same way to obtain more tar. We restricted the maximum number of tokens that each place can hold to one. This restriction is commonly used in workflow nets to ensure that time complexity was not a practical constraint for the calculation of the metrics^43^. Finally, causal logic determined the control flow of the models. When actions needed to be executed for a certain amount of time, the beginning and end were represented by start-and end-activities, with a place in between denoting ‘in progress’.

The three production processes can be modeled with different levels of abstraction. In this study, a specific level of abstraction was chosen to capture the structural complexity by employing basic computational task constructs. Additionally, the aim was to facilitate the mapping of crucial events that can be inferred from archaeological data through material analyses. For this reason, we employed coarse descriptions involving events that altered resource location or modified physical properties. Additionally, due to the limited availability of archaeological data for this ancient technology, it was necessary to rely on expert domain knowledge and insights. The collaboration with subject specialists deeply familiar with the modeled systems helped determine the achievable abstraction level, capturing essential dynamics and relationships while minimizing unnecessary complexity. Tar production methods were modeled as distinct systems. We chose this approach because these methods might have been utilized simultaneously or at different points in the past. Given their distinct nature, achieving complete equivalence in the complexity of their transitions is challenging. To make the transitions as consistent as possible, we focused on leveraging the causal relations between events and imposing constraints on the models. These constraints allowed us to implement the metrics discussed in the text. By adhering to these principles, our aim was to establish a dependable and consistent representation of the modeled processes. It is important to note that, to comply with the formal structure of workflow nets and to accurately represent the concurrent nature of activities during the process, we added the ‘preparations’ transition to all models. This addition was necessary to ensure the soundness of the models and to enable the application of the metrics.

The Petri nets were saved as pnml files (see Supplementary text S1), an XML-based interchange format for Petri nets, using Snoopy 2 version 1.22^46^. To analyze the Petri net models, we calculated the three metrics using ProM Tools release 6.10 developed by the Process Mining Group at Eindhoven Technical University^47,48^. We imported pnml files produced in Snoopy to ProM 6.10 using the plug-in PNML Petri net files. We used the plug-in Petri-net Metrics in ProM 6.10 for calculating the metrics. Reachability graphs were also calculated using ProM 6.10.

We utilized ProM 6.10 to perform a simple stochastic simulation of the Petri nets to generate 1000 synthetic runs of events (traces). In the simulations, an event represented the execution of one of the transitions within each tar production model. Each trace represented a sequence of events (transitions) executed to obtain the tar product. These sequences were restricted to a maximum of 10,000 events per trace. All synthetic sequences can be found as Supplementary Table S2 online. During the simulations, all transitions were assigned equal weight, and in cases of conflict, they were executed randomly. We calculated confidence intervals for the mean number of events per production method at 80%, 95%, and 99% levels to assess the statistical significance of the results. By utilizing these logs, we approximated the behavioral aspects of the execution of the production methods.

Based on the nature of the processes being modeled, there may be some variation in the resolution of the transitions. To evaluate this potential bias in transition complexity, we computed the proportion of transitions that represented embedded components (composite transitions) relative to the total number of transitions within the Petri nets folded as state machine constructs. These folded nets resulted from the final iteration of the structuredness algorithm (see below), before folding all net transitions into a single transition. These nets showed composite transitions signifying embedded sequences, 'while' loops, and marked graphs. Additionally, there were other transitions that could not be simplified further into distinct components. We interpreted a high percentage of composite transitions as an indicator of high resolution, whereas a lower percentage suggested low resolution in the transition complexity of the models. Differences in the percentages of composite transitions between the models were taken as indication of the magnitude of the differences in the complexity of the modeled transitions.

### Metrics

Our analysis is centred on comprehending the structure and state space using the potential states and transitions of each tar production method. This direction was chosen because grasping the inherent comprehensibility of a process holds significant relevance for studying the process's invention, discovery, or execution. Similar approaches implement modeling and identification of common control flow patterns to evaluate the comprehensibility and cognitive complexity of process models^27,49,50^. The analysis of individual usage behavior from human subjects could be effectively conducted through the use of the Automatic Mental Model Evaluation method (AMME)^45^. However, such an investigation lay beyond the scope of our present study. To analyze structural properties and control flow patterns, we used three metrics commonly applied in the literature to measure the complexity and comprehensibility of process models.

**Density metric**. The density metric measures the degree of connection between events (transitions) and conditions/resources (places) in the process^42^. It can be used as a proxy for how much information a maker has to access when several actions can be executed at the same time or when several conditions are needed to execute a given action in the process. The density metric in a workflow net calculates the ratio of existing arcs to the maximum number of possible arcs. The maximum number or arcs is calculated by multiplying the total number of places and transitions and then multiplying the result by two. A high ratio of existing arcs means that conditions/resources and actions are more interconnected, and therefore the amount of information required to change states of the process is higher. For instance, in Fig. 1, the Petri net has six places and four transitions, which implies that the maximum number of arcs possible is 48. The actual number of arcs present in the Petri net is 10. As a result, the density metric value for this Petri net is calculated to be 0.208.

**Extended Cyclomatic metric**. The extended cyclomatic metric measures the number of possible paths through which the final product can be obtained given the structure of the modeled system^43^. This metric is an extension of the cyclomatic metric^51^ which has previously been validated to measure cognitive complexity of human individuals^52^ We use it as a proxy for the likelihood of errors and reassessments throughout the process. The extended cyclomatic metric is calculated using the reachability graph of a Petri net model. This graph identifies all the possible moments (reachable states) at which a production system can be observed before obtaining the product^17^. In the reachability graph, reachable states can be represented as ovals (vertices), and the events that change the system’s states are represented as arrows (edges) connecting the ovals. The number of strongly connected components of each reachability graph is considered in the calculation. These components are subsets of reachable states of the reachability graph, where each state can be reached from any other state in the component. The minimal strongly connected component is represented by a single reachable state. The extended cyclomatic metric is calculated by subtracting the total number of vertices (reachable states) from the total number of directed edges and adding the number of strongly connected components to the result^43^. Practically, this means that low values in the extended cyclomatic metric imply fewer possible paths to reach the product, and lower chances of producing errors. High values mean that several different paths exist which translates to more possible errors during the process which may generate higher system complexity. In Fig. 1, there are six vertices (a, b, c, d, e, f), six edges (thick arrows), and six strongly connected components because all reachable states are minimal strongly connected components. As a result, the extended cyclomatic metric value for the reachability graph in Fig. 1 is six.

**Structuredness metric**. The structuredness metric measures the effort required to understand the information behind the process^43^ by deconstructing the Petri net models into components similar to programming constructs such as sequences, selections, and iterations^35,53^. These constructs are given a weight based on their structural complexity, which is a reported factor that reduces the comprehension of conceptual models^43,54^. In Petri nets, these constructs are sets of places, transitions, and arcs. Constructs with a smaller number of elements allow the information about the process to be transmitted easier and more accurately. This makes it easier to store information about components for future use, meaning the process is easier to learn. The algorithm to calculate the metric searches for seven types of components: (1) sequence, (2) choice, (3) while, (4) marked graph, (5) state machine, (6) well-structured, and (7) unstructured^40,53^. The metric is calculated using a function that weighs each component following the order above. First, the algorithm searches for sequences; the less complex components which receive the lowest weight, and it finishes with unstructured components, which receive the highest weight. Next, a weight is calculated for the identified component which is folded into a single transition. Then, the algorithm continues to search, weight, and fold components in the Petri net using the same priority function. This procedure continues until only a single transition remains, which is connected to both an input place and an output place. The weight of the last transition represents all the weights of the identified components. The metric gives higher weights to components containing embedded components, based on the assumption that components made of other nested components are more complicated and therefore harder to understand. A low metric score means a process has low complexity because the amount of information embedded in its structure is small. A high value indicates that the amount of information is large and it will require more effort to understand the structure of the process. For instance, when we use the algorithm to calculate the structuredness metric of the Petri net in Fig. 1, the only component found is a marked graph. The weight function for this component is determined by doubling the number of transitions and multiplying it with the *diff* function, which measures how evenly split and merge points are matched within the component. A higher *diff* value indicates unevenness in a workflow net. The marked graph in Fig. 1 has four transition and a *diff* value of one, making the structuredness metric value equal to eight. Please refer to Lassen and van der Aalst^43^ for further details on the weight functions and algorithm.

## Results

**Figure 2:**
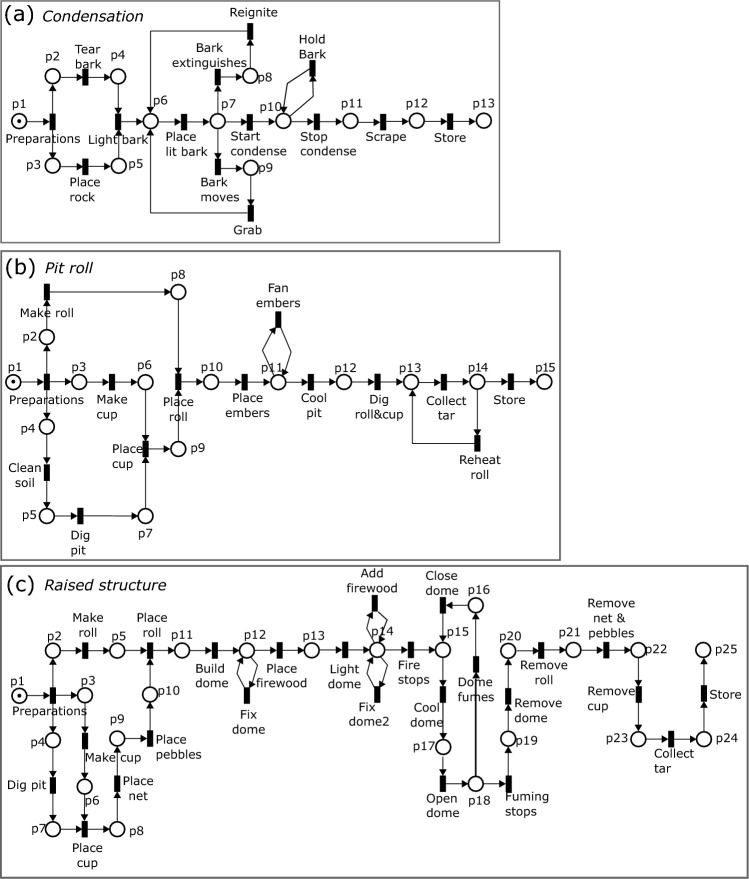
Initial state of Petri net models for the three tar production methods. *Condensation* (**a**); *pit roll* (**b**); *raised structure* (**c**). Places (circles) represent the conditions or resources before or after an event. Transitions (rectangles) represent events that change the local states of the system. Arcs (arrows) are directed and form logical connections between places and transitions. They indicate the flow of the system and the causal relations between places and transitions. Tokens inside places (black dots) represent availability of resources or the fulfillment of conditions.

A comparison of the three workflow nets showed that the *condensation* and *pit roll* methods have fewer conditions (places), events (transitions) and relations (arcs) than the *raised structure* method. The *condensation* model has the smallest number for all elements (Fig. 2a, Table 1). The *pit roll* model is slightly larger in each element category (Fig. 2b, Table 1), and the *raised structure* model is the largest in all categories (Fig. 2c, Table 1). In each net, places represent approximately 24%, transitions 24%, and arcs 52% of all elements. Our findings reveal that both the *condensation* and *raised structure* models demonstrate comparable levels of transition resolution and complexity. Within the nets folded as state machine constructs, approximately 60% of the transitions were associated with composite transitions for the *condensation* model, while the *raised structure* model showed a similar proportion of 57% (Table 1). In contrast, the *pit roll* model exhibited a lower percentage of 25% for composite transitions within the net folded as a state machine.

**Table 1:** Comparison of Petri Net elements, reachability graphs, structuredness constructs, and components of Petri nets folded as state machine constructs in Tar Production Models.

	Condensation	Pit roll	Raised structure
**Petri net models**			
Places	13	15	25
Transitions	14	14	26
Arcs	30	32	56
**Reachability graph**			
States	13	21	30
Edges	16	32	41
Strongly connected components	10	20	27
**Components Structuredness algorithm**			
Sequences	4	3	12
Whiles	1	1	1
Marked Graphs	1	1	1
State machine	1	1	1
**Components of Petri nets folded as state machine constructs**			
Composite transitions	3	1	4
Total transitions	5	4	7
Places	4	4	5
Percentage composite transitions	60%	25%	57%

**Figure 3:**
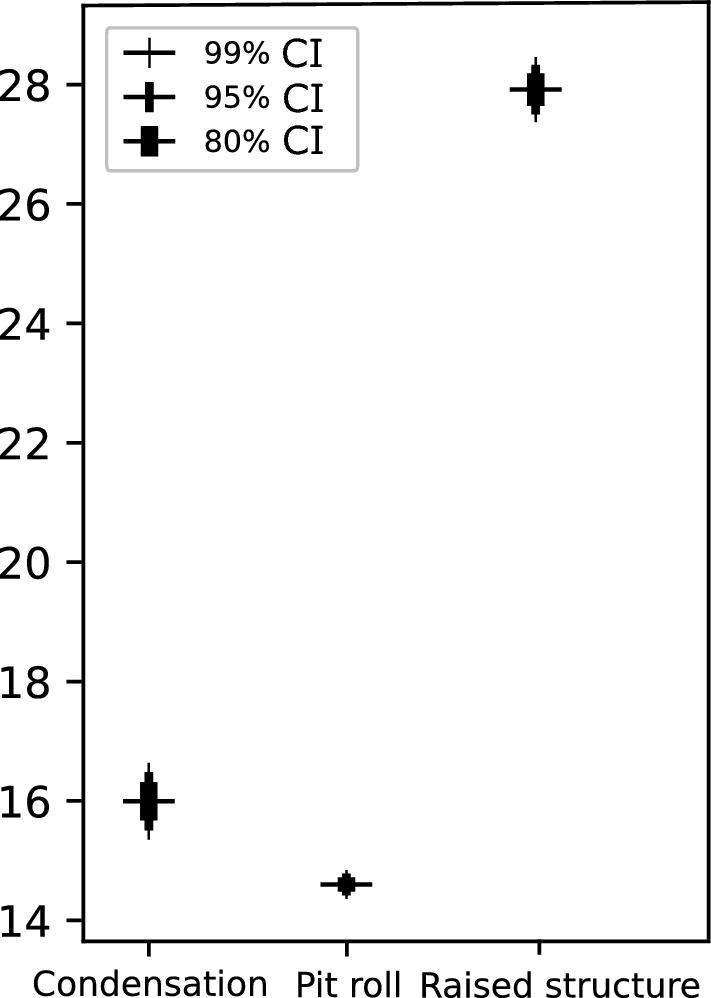
Comparison of confidence intervals (CI) at 80%, 95%, and 99% level for the mean of events in simulated traces of the *condensation*, *pit roll*, and *raised structure* models.

In the simulations (Table 2), the *pit roll* model showed the fewest events on average (mean = 15, min = 12, max = 32, standard deviation = 3), followed by the *condensation* (mean = 16, min = 9, max = 66, standard deviation = 8), and finally the *raised structure* (mean = 28, min = 21, max = 75, standard deviation = 7). The differences in the mean number of events between the three models remain statistically significant even when considering a 99% confidence interval (Fig. 3).

**Table 2:** Simulations results and descriptive statistics of the events per trace for the three tar production models.

	Condensation	Pit roll	Raised structure
**Simulations**			
Traces	1000	1000	1000
Total events	16,220	14,826	28,291
Trace variants	311	262	681
**Events per trace**			
Mean	16	15	28
Standard deviation	8	3	7
Min	9	12	21
25th percentile	10	12	23
50th percentile	14	14	27
75th percentile	19	16	31
max	66	32	75

### The condensation method requires more processing of information at the same time

**Figure 4:**
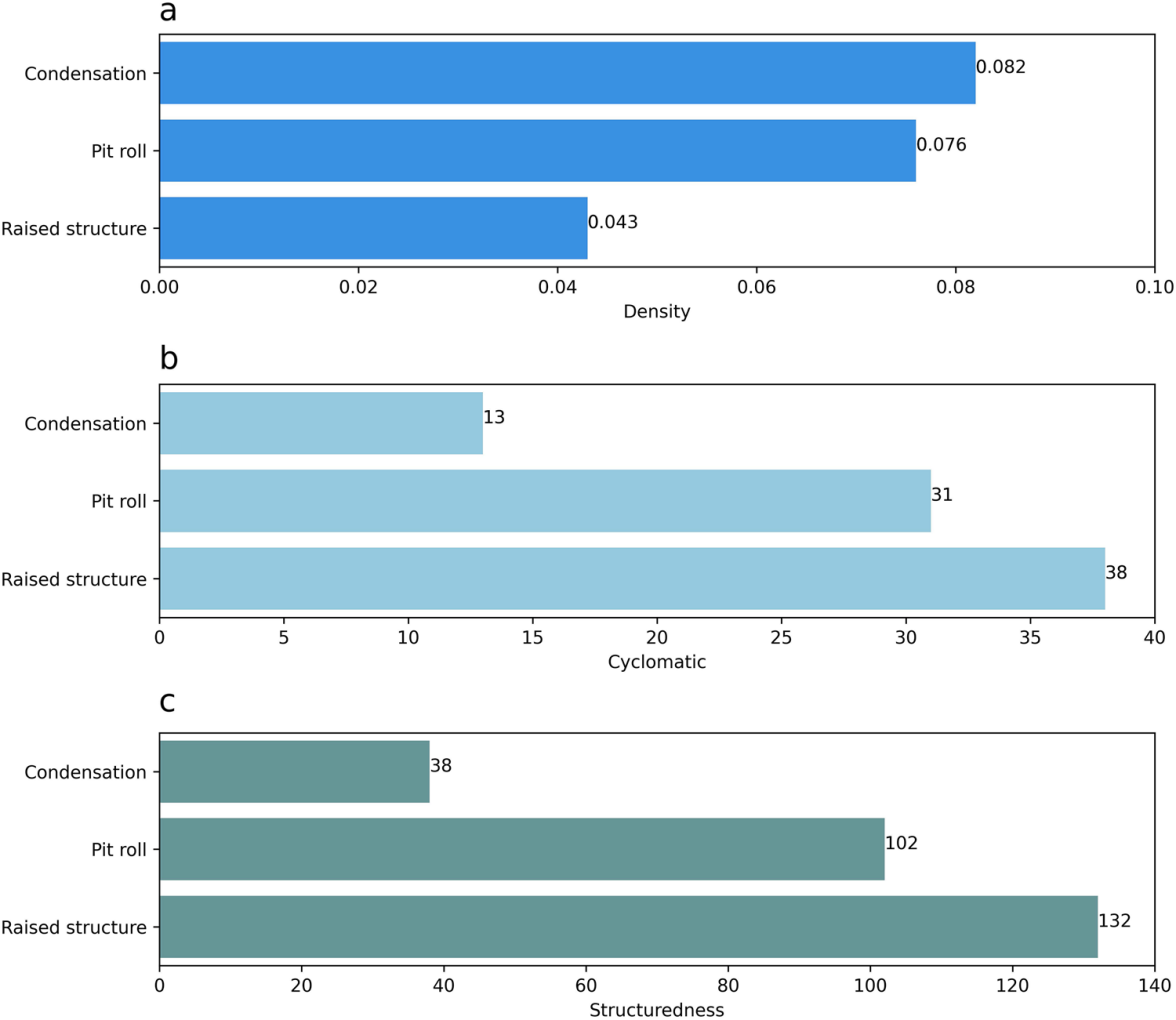
Complexity metrics values for the models of tar production. Density (**a**), Extended Cyclomatic (**b**) and Structuredness (**c**).

The *condensation* model scored the highest in the density metric (*value* = 0.082), followed by the *pit roll* model (*value* = 0.076), and then the *raised structure* model (*value* = 0.043; Fig. 4a). The density value of the *condensation* model can be explained by the information processing peak during the use of fire. Considering the number of possible connections between transitions and places, and the size of the model, the density metric shows that this peak is more demanding than those in the other models. The use of fire is modeled with the transition ‘Light bark’, and the place ‘p6’. These elements are connected with other transitions and places of the model by three and four arcs, respectively (Fig. 4a). The arc density is also higher during and after using fire in the *condensation* method than in the *pit roll* and *raised structure* methods. Nine arcs are located before the transition ‘Light bark’, and 21 arcs occur after in the *condensation* model.

The *pit roll* model scored the second highest density of the three models (*value*= 0.076). When compared with the *condensation* model, this value results from a larger number of conditions to be fulfilled without a strong increment in the arc density. The *pit roll* model included only two more arcs and two more places than the *condensation* model. Three transitions and three places are connected each with more than two arcs (Fig. 4c). Nineteen arcs in the *pit roll* model are located before the start of use of fire, represented by the transition ‘Place embers’, and the other 13 arcs appear after. This net structure shows that the arc density and the number of conditions are higher in preparations before using fire than during the use of fire.

The *raised structure* model showed the lowest density (*value* = 0.043). There are four places and three transitions in the Petri net connected with more than two arcs with other elements (Fig. 4c). However, the *raised structure* model almost doubles the number of places, transitions, and arcs compared with the other two models. This reduced the weight that the multiple arcs have in the density. When comparing net structures of the *raised structure* and *condensation* models, the *raised structure* model shows arcs that are more uniformly distributed between the preparations before using fire and the actions involved in the use of fire. Twenty seven arcs are located before the use of fire, represented by the transition ‘Light dome’, and 29 arcs appear after. The *raised structure* model is therefore balanced in terms of the actions associated with the preparations before using fire and the actions during and after the use of fire.

### The pit roll and raised structured methods require control actions to reduce errors

**Figure 5:**
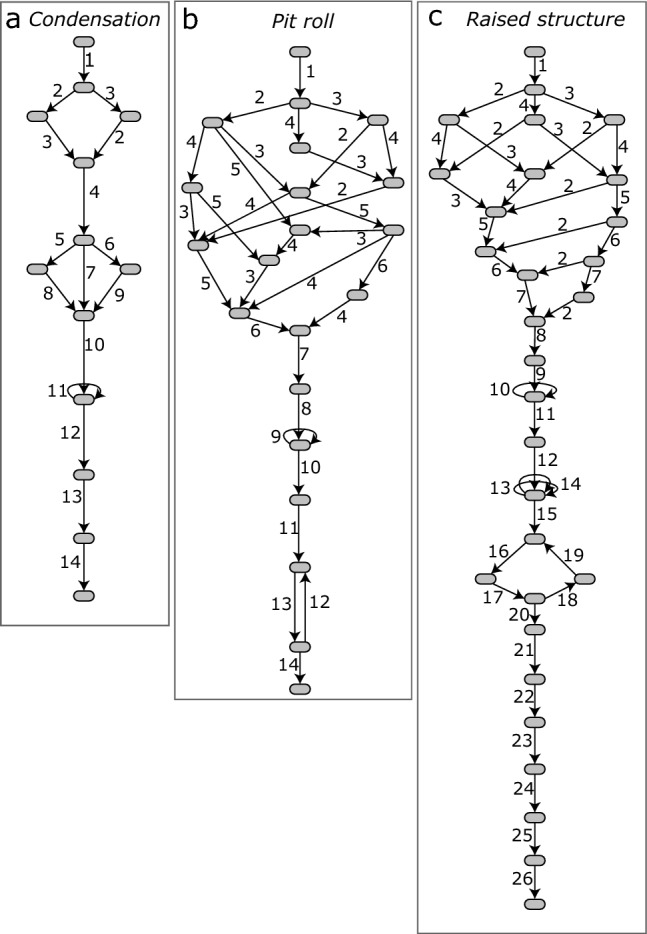
Reachability graphs showing states (vertices) and transitions (numbered edges) for each tar production model. (**a**) *Condensation* (1. Start, 2. Tear bark, 3. Place rock, 4. Light bark, 5. Grab, 6. Reignite, 7. Place lit bark, 8. Bark moves, 9. Bark extinguishes, 10. Start condense, 11. Hold bark, 12. Stop condense, 13. Scrape, 14. Store); (**b**) *pit roll* (1. Start, 2. Clean soil, 3. Make cup, 4. Make roll, 5. Dig pit, 6. Place cup, 7. Place roll, 8. Place embers, 9. Fan embers, 10. Cool pit, 11. Dig roll & cup, 12. Reheat roll, 13. Collect tar, 14. Store); (**c**) *raised structure* (1. Start, 2. Make roll, 3. Dig pit, 4. Make cup, 5. Place cup, 6. Place net, 7. Place pebbles, 8. Place roll, 9. Make dome, 10. Fix dome, 11. Place firewood, 12. Light dome, 13. Add firewood, 14. Fix dome2, 15. Fire stops, 16. Cool dome, 17. Open dome, 18. Dome fumes, 19. Close dome, 20. Fuming stops, 21. Remove dome, 22. Remove roll, 23. Remove net & pebbles, 24. Remove cup, 25. Collect tar, 26. Store).

The *condensation* model scored the lowest in the extended cyclomatic metric (*value* = 13), followed by the *pit roll* model (*value* = 31), and then the *raised structure* model (*value* = 38, Fig. 4b). A comparison of the number (Table 1) and distribution (Fig. 5) of directed edges, vertices and strongly connected components in the reachability graphs provide insights into the factors generating the differences in this metric.

The low score of the *condensation* model (*value* = 13), and the low number of reachable states are explained by the number of transitions that can be executed concurrently and the small number of sequential actions. Thirty eight percent (N=5) of the total reachable states occur during the preparations before the use of fire. The only two transitions (‘Tear bark’ and ‘Place rock’) that may occur concurrently at any given marking are part of the preparations. The potential for concurrent actions in the *condensation* model is the lowest of the three models. The largest strongly connected components in the *condensation* model has four reachable states representing 30% of the total. This is the highest percentage for any strongly connected component in the three models. The largest strongly connected component in the *condensation* model occurs after the transition ‘Light bark’ (Fig. 5a). One repetitive action (‘Hold bark’) occurs as a self-loop during the use of fire.

The *pit roll* model scored the second highest for the extended cyclomatic metric (value=31). Compared with the *condensation* model, the reachability graph of the *pit roll* model has almost double the number of reachable states, and twice the number of edges and strongly connected components (Fig. 5b; Table 1). Seventy six percent (N=16) of the total reachable states occur during the preparations for the use of fire, where a maximum of three transitions in any combination from the set ‘Make cup’; ‘Clean soil; ‘Dig pit’; ‘Place cup’ and ‘Make roll’ can occur concurrently (Fig. 2b). The maximum number of possible concurrent transitions in the *pit roll* suggests that concurrent actions are more important in the *pit roll* method than in the *condensation* method. The largest strongly connected component in the reachability graph of the *pit roll* model has two reachable states and represents 9.5% of the total. One repetitive action (‘Fan’) occurs as a self-loop during the use of fire.

The *raised structure* model scored the highest for the extended cyclomatic metric (value= 38). The *raised structure* model is the longest of the three models and also shows the largest number of sequences (Fig. 5c; Table 1). This model also includes transitions with the potential to be executed concurrently before the use of fire, and repetition of actions during the use of fire. Fifty three percent (N=18) of the total reachable states occur during the preparations for the use of fire. A maximum of three of the transitions ‘Make cup’, ‘Place net’, ‘Place pebbles’, ‘Dig pit’, ‘Place cup’, and ‘Make roll’ can occur concurrently in any combination (Fig. 2c). The largest strongly connected component in the reachability graph of the *raised structure* model has four reachable states and represents 13% of the total. This strongly connected component, generated by a cycle of actions during the opening of the structure, is twice the size of that from the *pit roll* and has the same number of reachable states as the *condensation* model. Three other actions (‘Fix dome’; ‘Add firewood’; ‘Fix dome 2’) are repeated as self-loops, separated by sequences of actions during the use of fire.

### The raised structure contains more embedded information than the condensation or pit roll methods.

The *condensation* model scored the lowest in the structuredness metric (value = 38), followed by the *pit roll* (value = 102), and then the *raised structure* (value = 132; Fig 3C). The metric values indicate that the *raised structure* model requires more planning and acquiring more knowledge about the production process than the *pit roll* and *condensation* models do. All three Petri net models showed sequences, whiles, marked graphs, and state machines (Table 1), organized in a three-tier hierarchical structure. Sequences are found in the deepest tier inside the marked graph of the *pit roll* and *raised structure* models, but they are also found embedded in the state machine components in the second tier in all nets. Sequences are the most dominant component representing 68% of all components matched. The *raised structure* model shows the highest number of sequences (N = 12) and the *pit roll* model the lowest (N = 3).

The while components appear in the second tier of the deconstructed nets, embedded within the state machine components. Each model contained one while component.

The marked graph components were found with different sizes in the second tier of the deconstructed nets. The marked graphs relate to the preparations before the use of fire. The marked graph of the *condensation* model is the smallest, collapsing four places and four transitions. The marked graph of the *pit roll* model collapses eight places and seven transitions, and has one embedded sequence. The *raised structure* model has a marked graph that collapses nine places and has two embedded sequences.

The top tier of the hierarchical structure of the decomposed nets is a state machine component. The sequences, whiles and marked graphs are embedded in this tier. The top tier shows different sizes for each model with composite transitions representing the embedded sequences, whiles, and marked graphs described above. At the top tier of the folded net Table 1, the *condensation* model had a total of four places, three composite transitions, and two transitions. The *pit roll* model showed four places, one composite transition, and three transitions. Finally, the *raised structure* model showed five places, four composite transitions, and three transitions.

## Discussion

The experiments, the Petri net models, and the metrics show that the *condensation* method primarily requires a mechanism to process information at the same time. The complexity metrics also show that the *raised structure* method relies on cognitive functions that combine the use of different cognitive processes, such as information processing, planning, and learning. The simulations showed longer sequences of events for the *raised structure* and the *condensation* model, indicating a higher expected behavioral complexity in real-world scenarios. However, it is also worth noting that as expertise of the maker is developed over time, it may reduce the length of the observable behavior duration during product production^55^.

Based on the structure of the Petri net models and the density metric, the *condensation* method imposes the most interconnection between elements of the process of all three methods (Table 1). The values of the density metric show that actions and resources are more interconnected in the *condensation* model. Having more actions and resources interacting at the same time requires accessing more information at a given time. The cognitive load in the *condensation* method is generated by the attention required to maintain the pieces of lit bark before and during the tar condensing on the cobble. The results for the *pit roll* model indicate that it requires less attention than the *condensation* model and that the most interconnected elements and actions occur during the preparations before using fire. Any mechanism used to process information at the same time would be less intensely used in the *pit roll* and *raised structure* methods because peaks in information processing are smoother compared with the *condensation* model. In the *raised structure* method, actions are less interconnected and more distributed through the entire process, so the focus of the maker can be on fewer actions at a time. The intensity and allocation of attention are two modern human cognitive resources that allow us to process and navigate through large amounts of information^56^.

The lower likelihood of errors shown in the cyclomatic metric suggests that the *condensation* method is a simpler solution because fewer possible paths exist to obtain the tar product. This means that the variability in the way the process works, and thus the underlying system is less complex than in the *pit roll* and *raised structure* methods. The *pit roll* model is similar to the *raised structure* in that it shows more possible paths and a higher likelihood of errors than the *condensation* model. The potential for concurrency generates most of the system complexity of the *pit roll* method. The results show that the possible paths to reach the final product and the possibility of errors of the *raised structure* model are generated by combinations of concurrent activities, actions executed as cycles, and repetitive actions executed as self-loops. The *raised structure* has the largest number of reachable states, paths, and possible errors to reach the final product.

The *condensation* method is the only method where fewer reachable states are present before fire than after (Figs. 2, 5). The results show that the system complexity in the *condensation* model after the use of fire is associated with repetitive actions that may occur as cycles or self-loops. The potential for concurrency and the largest number of reachable states of the *pit roll* and *raised structure* methods occurs before fire, suggesting that the technical events during preparation are more cognitively demanding. The use of fire is unique among actions in tar production. No possible concurrent actions occur after the use of fire in any of the three models, meaning that fire use is a synchronization event where all tasks that can be conducted concurrently come together, and attention shifts from being divided among multiple possible actions, to being focused on one action at a time. This is done to finish the process and obtain the product. The need for synchronization of material flows obtained via concurrent events is a feature that appears in human made systems. For example, in ceramic production systems that mix clays to obtain pottery with specific properties^57^.

The models and the structuredness metric suggest that the *condensation* method is easier to learn than the two other methods. The embedded components of the *condensation* model, and especially its marked graph, are smaller, suggesting that the amount of information required to execute the process is also smaller. The *condensation* model scored the lowest in the structuredness metric, despite having more transitions in its state machine component than the *pit roll* model. The marked graph of the *pit roll* model is the second largest and has more actions with potential for concurrent activities than the *condensation* model, making the score of the *pit roll* model the second highest in the structuredness metric. The *raised structure* model scored the highest in the extended cyclomatic metric because it has more than twice the number of sequences in the workflow and its marked graph and state machine components are larger than the other two models. This suggests that the *raised structure* method requires more effort to understand the elements in the production process because it shows a more elaborated and larger process structure than the *pit roll* and *condensation* models. The results also indicate that the *pit roll* and *raised structure* model have at least three times more embedded information in their process structures than the *condensation* model. The *raised structure* model shows the largest amount of information in its structure, meaning that greater understanding and planning is needed to complete the process. The experiment observations further support this and showed that the *pit roll* and the *raised structure* methods required more planning for their execution. Differences in the number of embedded transitions suggest that the *pit roll* model was abstracted at a lower resolution compared to the *condensation* and *raised structure* models. As a result, exercising caution is recommended when interpreting the differences in magnitude of metric values derived for the *pit roll* model in direct comparison to those of the *raised structure* and *condensation* models. Nevertheless, the differences in understandability of the three methods show that reasoning and planning, common cognitive processes used by modern humans^56,58^, may have been involved in some of the tar production methods available to Neanderthals.

Recent studies show evidence of production processes by Neanderthals like plant cooking^59^, fire use^60^, and tar production^18,61^ with multiple steps and components that likely developed over time. Our findings indicate that regardless of the methods employed, prehistoric tar making likely demanded a level of information processing that extended beyond simple behaviors. Instead, it appears to have involved a form of technical cognition akin to that observed in modern humans, and probably employed in comparable ways^2,62,63^ (but see also^64^). This is observed in similarities regarding the amount of information and the number of conditions needed to execute each of the three methods. The capacity of working memory for contemporary humans is estimated at around four items^65^, but recent studies show that working memory usage maintains two to three features simultaneously^66^. In the *condensation* method, the peak of conditions that need to be dealt with at the same time has three components: the positioning of the bark against the rock, the condition of the ignited bark, and the initiation of *condensation*. The attention required to monitor these three conditions creates the relations between resources and activities that gives the *condensation* method its high-density metric value. Moreover, a maximum of two concurrent activities in the *condensation* method, and three activities in the *pit roll* and *raised structure* methods need to be taking into account at the same time to avoid repetition. This is also required for components of composite tools that are produced asynchronously^39,67^. These results suggest the need for systematic testing of cognitive requirements and the differences in how Neanderthals and early modern humans developed their technologies. The method presented here, which involves analyzing three different aspects of a production process, facilitates such comparisons for future studies.

The three production processes all contain choices that require inhibitory control. To ensure that the process will produce tar when its execution terminates, makers are required to control their need of obtaining tar. All the models ensure termination of the production processes and the cyclomatic metric evaluates every possible combination of actions in modeled production processes. However, in real world situations, we cannot ensure that a production process will yield the desired product if it terminates because not all events will occur successfully every time. Interference, random events, or urgency in obtaining the product may prevent makers from fetching tar, even if all steps in the process are executed. For example, opening the dome of the *raised structure* without giving enough time to reach high temperatures increases the possibility of not obtaining tar, even if all the steps in the process are executed. Therefore, self-control is required in tar production. This type of self-control in the production of material culture is argued to date back to the Early Stone Age and is a prerequisite for any tool-making and extended problem solution distance behaviors^4,63,68^. For example, in stone-tool making^69^ and specifically in the production of symmetrical Acheulean handaxes^70^ self-control is required to invest time to acquire the required stone knapping skills. The modeling approach and the metrics presented here can be helpful to test such hypotheses in the future.

If the information required to produce a technology is acquired cumulatively^71,72^ (but also see)^73^, processes with low understandability (i.e., more information), such as the *raised structure*, are likely to emerge later in the development of a technology. Conversely, processes with high understandability are likely to be more prevalent during the emergence of a technology. In this light, the *condensation* method could have been discovered first^15^. It relies on materials directly available in the environment and the process of tar formation can be directly observed in an open fire. This technique may even qualify as a latent solution^15,74,75^; presented under the right circumstances to an individual and not requiring teaching. For Neanderthal tar making via *condensation*, these circumstances must have included the ability to synchronize information from distinct parts of the process, access to birch bark, a suitable rock, a tool for scraping, and fire. The other production methods have more embedded information, making them more difficult to learn. They are, therefore, unlikely to be latent solutions. It is more likely that, if used, the technological know-how of the *pit roll* and *raised structure* techniques were transmitted culturally^76^. These two production methods also rely on a greater planning depth and inhibition ability, and the integration of these with other cognitive processes.

Our study and method are not without limitations. Here we studied a single technology using three possible production methods.In reality, Neanderthals may have used more or different methods^18,77,78^. The results are by no means a complete representation of the complexity of the Neanderthal technological world; this would require modeling of the production of bone, wood, and stone tools, as well as other technologies like fire^79–84^. Moreover, for a comprehensive model of early technological complexity and its relation with cognitive evolution, comparisons require the inclusion of early modern human ancient technology.

In addition, with Petri nets we model a reconstructed version of the past; missing details in this reconstruction may influence measured outcomes. We were unable to incorporate additional factors of cognitive complexity, such as task complexity, understood as the minimum required problem-solving knowledge for a given task, and behavior complexity, referring to the observed behaviors employed to accomplish a task^52,85^. Instead, our approach centered on assessing the structure and state space within each tar production process. This method, based on the examination of possible states and transitions, was the most dependable proxy for our analysis.

The metrics used are designed for modern human processes and their relevance to ancient cognition may be questioned. In this study we work from the assumption that both Neanderthals and modern humans share similar evolutionary traits, and we therefore considered the metrics suitable. Basic metrics, such as counts of places, arcs, and transitions, can be influenced by the modeling language and process length. Also, composite complexity metrics such as the ones presented in our study, encounter difficulties when dealing with highly unstructured process models, a prevalent occurrence in ancient contexts. To reduce the impact of these biases, we employed multiple metrics in our analysis of the aceramic tar production process formalizations. We believe this methodological approach reduces any possible bias in the systematic analysis of technological production using process modeling within archaeology.

In future works, all known production methods of multiple technologies from ancient technological systems should be compared to illuminate the trends in technological and cognitive solutions available in the past. For some technological processes, like lithic where refits exist, much data may be present, whereas for others, like basketwork, there is heavy reliance on experimental archaeology. In addition, some of the tar production techniques are scalable and we did not model for that here. The models and metrics have limitations to study the role of division of tasks, cooperation^17^, language development in the production process, or the effects of resource availability in the process complexity. Other classes of Petri nets such as place/transitions nets are more adequate to explore these problems^17^.

Our focus was directed towards discerning the differences between methods based on possible states and transitions, rather than emphasizing actual usage by human participants. Testing the use of cognitive resources in real life scenarios could be approximated for the middle Paleolithic with a large experimental setting. However, that is outside of the scope of this study. Future research may use task sequential data from empirical scenarios involving participants with varying degrees of expertise in tar production. These records may be compared with the results for the top-down models presented in this study. The AMME toolkit^45^ is a valuable source to facilitate such comparison, particularly when it comes to understanding the cognitive complexity inherent in actual usage through the application of the Petri net formalism.

There is an increasing argument that Neanderthals possessed technological and cognitive capabilities comparable with modern humans^1,60,86,87^. Evidence from various Neanderthal technologies and other behaviors such as deep cave activity^88^, cave painting^89^, use of jewelry and body painting^90^, and deliberate burial^91^ have identified over the last decades. In our study, we have demonstrated the possibility of approximating the cognitive requirements of these technologies and behaviors. The use of Petri nets and their derived measures is particularly valuable, as they allow for comparisons of complexity across different materials and technologies. Furthermore, Petri nets hold promise as a tool for investigating older technologies, where making inferences is more challenging compared to the Middle Paleolithic period. This approach can shed light on the cognitive abilities of our earliest tool-making ancestors.

## Conclusion

The method presented here links process complexity of ancient technologies to cognitive processes. At face value the *condensation* method requires more simultaneous processing of information than the other two methods. However, the two other methods are more demanding in terms of knowledge density, and require cultural learning, self-control, and planning. The Petri net approach to measure complexity presented here has proven to be useful for the systematic analysis and comparison of ancient technologies and their production systems. Independent of which proposed tar production methods were used in the past, the Petri net models and complexity metrics suggest that Neanderthals probably relied on several cognitive traits that archaeologists often associate with modern behavior. Our multi-angled approach to the embedded information in technological processes does justice to the different and idiosyncratic forms of technological complexity and cognitive traits hominins may have had. Future studies can extend this implementation to other technological processes to improve our understanding of human and technological evolution.

### Supplementary Information

Supplementary Information 1.

Supplementary Information 2.

## Data Availability

All of the study data are included in the article or in the supporting information.
