# Supplementary material for: Measuring ancient technological complexity and its cognitive implications using Petri nets
Source: Sci Rep. 2023 Sep 22;13:14961. doi: 10.1038/s41598-023-42078-1 (PMC10516984; doi:10.1038/s41598-023-42078-1)
Supplement: Supplementary file 1 — Supplementary Information 1. [file 41598_2023_42078_MOESM1_ESM.pdf]

Supplementary Information for

Measuring ancient technological complexity and its cognitive implications using Petri nets

Sebastian Fajardo, Paul R. B. Kozowyk, Geeske H. J. Langejans

Sebastian Fajardo
E-mail: s.d.fajardobernal@tudelft.nl

This PDF file includes:

Supporting text
SI References

Supporting Information Text

Models

Here we provided the plain text versions of the Petri net models in PNML format. Each model's plain text is structured within the corresponding subsection in an XML-based interchange format. You can save each section as a .pnml file using any text editor. These pnml files can be read by various tools including Snoopy 2 version 1.22 (1). and Prom 6.10 (2, 3).

To replicate the results, you can use Prom 6.10. Imported each PNML file as a separate file using the plug-in PNML Petri net files. Then you can employ plug-in Petri-net Metrics to calculate the metrics. Additionally, Prom 6.10 provides the plug-in Petri net to Reachability Graph to generate the reachability graph for each model.

A. Condensation model.


```
<?xml version="1.0" encoding="UTF-8"?>
<pnml xmlns="http://www.pnml.org/version-2009/grammar/pnml">
  <net id="net0" type="http://www.pnml.org/version-2009/grammar/ptnet">
    <name>
      <text>_20210424_condensation09_workflow_V1_SF_pn</text>
    </name>
    <page id="page0">
      <name>
        <text>DefaultPage</text>
      </name>
      <place id="p0">
        <name>
          <text>p1</text>
        </name>
        <initialMarking>
          <text>1</text>
        </initialMarking>
      </place>
      <place id="p1">
        <name>
          <text>p2</text>
        </name>
        <initialMarking>
          <text>0</text>
        </initialMarking>
      </place>
      <place id="p2">
        <name>
          <text>p3</text>
        </name>
        <initialMarking>
          <text>0</text>
        </initialMarking>
      </place>
      <place id="p3">
        <name>
          <text>p4</text>
        </name>
        <initialMarking>
          <text>0</text>
        </initialMarking>
      </place>
      <place id="p4">
        <name>
          <text>p5</text>
        </name>
        <initialMarking>
          <text>0</text>
        </initialMarking>
      </place>
```

```
<place id="p5">
  <name>
    <text>p6</text>
  </name>
  <initialMarking>
    <text>0</text>
  </initialMarking>
</place>
<place id="p6">
  <name>
    <text>p8</text>
  </name>
  <initialMarking>
    <text>0</text>
  </initialMarking>
</place>
<place id="p7">
  <name>
    <text>p7</text>
  </name>
  <initialMarking>
    <text>0</text>
  </initialMarking>
</place>
<place id="p8">
  <name>
    <text>p9</text>
  </name>
  <initialMarking>
    <text>0</text>
  </initialMarking>
</place>
<place id="p9">
  <name>
    <text>p10</text>
  </name>
  <initialMarking>
    <text>0</text>
  </initialMarking>
</place>
<place id="p10">
  <name>
    <text>p11</text>
  </name>
  <initialMarking>
    <text>0</text>
  </initialMarking>
</place>
<place id="p11">
  <name>
    <text>p12</text>
  </name>
  <initialMarking>
    <text>0</text>
  </initialMarking>
</place>
<place id="p12">
  <name>
    <text>p13</text>
  </name>
  <initialMarking>
```

```
    <text>0</text>
  </initialMarking>
</place>
<transition id="t0">
  <name>
    <text>Store</text>
  </name>
</transition>
<transition id="t1">
  <name>
    <text>Scrape</text>
  </name>
</transition>
<transition id="t2">
  <name>
    <text>Start_condense</text>
  </name>
</transition>
<transition id="t3">
  <name>
    <text>Bark_extinguishes</text>
  </name>
</transition>
<transition id="t4">
  <name>
    <text>Reignite</text>
  </name>
</transition>
<transition id="t5">
  <name>
    <text>Place_Lit_bark</text>
  </name>
</transition>
<transition id="t6">
  <name>
    <text>Bark_moves</text>
  </name>
</transition>
<transition id="t7">
  <name>
    <text>Grab</text>
  </name>
</transition>
<transition id="t8">
  <name>
    <text>Light_bark</text>
  </name>
</transition>
<transition id="t9">
  <name>
    <text>Place_rock</text>
  </name>
</transition>
<transition id="t10">
  <name>
    <text>Tear_Bark</text>
  </name>
</transition>
<transition id="t11">
  <name>
    <text>Preparations</text>
```

```
    &lt;/name&gt;
  &lt;/transition&gt;
  &lt;transition id="t12"&gt;
    &lt;name&gt;
      &lt;text&gt;Hold_bark&lt;/text&gt;
    &lt;/name&gt;
  &lt;/transition&gt;
  &lt;transition id="t13"&gt;
    &lt;name&gt;
      &lt;text&gt;Stop_condense&lt;/text&gt;
    &lt;/name&gt;
  &lt;/transition&gt;
  &lt;arc id="a0" source="p0" target="t11"&gt;
    &lt;inscription&gt;
      &lt;text&gt;1&lt;/text&gt;
    &lt;/inscription&gt;
  &lt;/arc&gt;
  &lt;arc id="a1" source="t11" target="p1"&gt;
    &lt;inscription&gt;
      &lt;text&gt;1&lt;/text&gt;
    &lt;/inscription&gt;
  &lt;/arc&gt;
  &lt;arc id="a2" source="t11" target="p2"&gt;
    &lt;inscription&gt;
      &lt;text&gt;1&lt;/text&gt;
    &lt;/inscription&gt;
  &lt;/arc&gt;
  &lt;arc id="a3" source="p1" target="t10"&gt;
    &lt;inscription&gt;
      &lt;text&gt;1&lt;/text&gt;
    &lt;/inscription&gt;
  &lt;/arc&gt;
  &lt;arc id="a4" source="p2" target="t9"&gt;
    &lt;inscription&gt;
      &lt;text&gt;1&lt;/text&gt;
    &lt;/inscription&gt;
  &lt;/arc&gt;
  &lt;arc id="a5" source="t10" target="p3"&gt;
    &lt;inscription&gt;
      &lt;text&gt;1&lt;/text&gt;
    &lt;/inscription&gt;
  &lt;/arc&gt;
  &lt;arc id="a6" source="t9" target="p4"&gt;
    &lt;inscription&gt;
      &lt;text&gt;1&lt;/text&gt;
    &lt;/inscription&gt;
  &lt;/arc&gt;
  &lt;arc id="a7" source="p3" target="t8"&gt;
    &lt;inscription&gt;
      &lt;text&gt;1&lt;/text&gt;
    &lt;/inscription&gt;
  &lt;/arc&gt;
  &lt;arc id="a8" source="t8" target="p5"&gt;
    &lt;inscription&gt;
      &lt;text&gt;1&lt;/text&gt;
    &lt;/inscription&gt;
  &lt;/arc&gt;
  &lt;arc id="a9" source="p5" target="t5"&gt;
    &lt;inscription&gt;
      &lt;text&gt;1&lt;/text&gt;
    &lt;/inscription&gt;
```

```
</arc>
<arc id="a10" source="p7" target="t3">
  <inscription>
    <text>1</text>
  </inscription>
</arc>
<arc id="a11" source="t3" target="p6">
  <inscription>
    <text>1</text>
  </inscription>
</arc>
<arc id="a12" source="p6" target="t4">
  <inscription>
    <text>1</text>
  </inscription>
</arc>
<arc id="a13" source="p7" target="t6">
  <inscription>
    <text>1</text>
  </inscription>
</arc>
<arc id="a14" source="t6" target="p8">
  <inscription>
    <text>1</text>
  </inscription>
</arc>
<arc id="a15" source="p8" target="t7">
  <inscription>
    <text>1</text>
  </inscription>
</arc>
<arc id="a16" source="t2" target="p9">
  <inscription>
    <text>1</text>
  </inscription>
</arc>
<arc id="a17" source="p4" target="t8">
  <inscription>
    <text>1</text>
  </inscription>
</arc>
<arc id="a18" source="t4" target="p5">
  <inscription>
    <text>1</text>
  </inscription>
</arc>
<arc id="a19" source="t5" target="p7">
  <inscription>
    <text>1</text>
  </inscription>
</arc>
<arc id="a20" source="t7" target="p5">
  <inscription>
    <text>1</text>
  </inscription>
</arc>
<arc id="a21" source="p7" target="t2">
  <inscription>
    <text>1</text>
  </inscription>
</arc>
```

```
      <arc id="a22" source="t0" target="p12">
        <inscription>
          <text>1</text>
        </inscription>
      </arc>
      <arc id="a23" source="p9" target="t12">
        <inscription>
          <text>1</text>
        </inscription>
      </arc>
      <arc id="a24" source="t12" target="p9">
        <inscription>
          <text>1</text>
        </inscription>
      </arc>
      <arc id="a25" source="p9" target="t13">
        <inscription>
          <text>1</text>
        </inscription>
      </arc>
      <arc id="a26" source="t13" target="p10">
        <inscription>
          <text>1</text>
        </inscription>
      </arc>
      <arc id="a27" source="p10" target="t1">
        <inscription>
          <text>1</text>
        </inscription>
      </arc>
      <arc id="a28" source="t1" target="p11">
        <inscription>
          <text>1</text>
        </inscription>
      </arc>
      <arc id="a29" source="p11" target="t0">
        <inscription>
          <text>1</text>
        </inscription>
      </arc>
    </page>
  </net>
</pnml>
```

B. Pit roll model.

```
<?xml version="1.0" encoding="UTF-8"?>
<pnml xmlns="http://www.pnml.org/version-2009/grammar/pnml">
  <net id="net0" type="http://www.pnml.org/version-2009/grammar/ptnet">
    <name>
      <text>_20210424_pitroll11_workflow_V1_SF_pn</text>
    </name>
    <page id="page0">
      <name>
        <text>DefaultPage</text>
      </name>
      <place id="p0">
        <name>
          <text>p1</text>
        </name>
```

```
    <initialMarking>
      <text>1</text>
    </initialMarking>
  </place>
  <place id="p1">
    <name>
      <text>p4</text>
    </name>
    <initialMarking>
      <text>0</text>
    </initialMarking>
  </place>
  <place id="p2">
    <name>
      <text>p3</text>
    </name>
    <initialMarking>
      <text>0</text>
    </initialMarking>
  </place>
  <place id="p3">
    <name>
      <text>p2</text>
    </name>
    <initialMarking>
      <text>0</text>
    </initialMarking>
  </place>
  <place id="p4">
    <name>
      <text>p6</text>
    </name>
    <initialMarking>
      <text>0</text>
    </initialMarking>
  </place>
  <place id="p5">
    <name>
      <text>p7</text>
    </name>
    <initialMarking>
      <text>0</text>
    </initialMarking>
  </place>
  <place id="p6">
    <name>
      <text>p9</text>
    </name>
    <initialMarking>
      <text>0</text>
    </initialMarking>
  </place>
  <place id="p7">
    <name>
      <text>p8</text>
    </name>
    <initialMarking>
      <text>0</text>
    </initialMarking>
  </place>
  <place id="p8">
```

```
  <name>
    <text>p10</text>
  </name>
  <initialMarking>
    <text>0</text>
  </initialMarking>
</place>
<place id="p9">
  <name>
    <text>p11</text>
  </name>
  <initialMarking>
    <text>0</text>
  </initialMarking>
</place>
<place id="p10">
  <name>
    <text>p13</text>
  </name>
  <initialMarking>
    <text>0</text>
  </initialMarking>
</place>
<place id="p11">
  <name>
    <text>p14</text>
  </name>
  <initialMarking>
    <text>0</text>
  </initialMarking>
</place>
<place id="p12">
  <name>
    <text>p5</text>
  </name>
  <initialMarking>
    <text>0</text>
  </initialMarking>
</place>
<place id="p13">
  <name>
    <text>p15</text>
  </name>
  <initialMarking>
    <text>0</text>
  </initialMarking>
</place>
<place id="p14">
  <name>
    <text>p12</text>
  </name>
  <initialMarking>
    <text>0</text>
  </initialMarking>
</place>
<transition id="t0">
  <name>
    <text>Dig_pit</text>
  </name>
</transition>
<transition id="t1">
```

```
    <name>
      <text>Make_cup</text>
    </name>
  </transition>
  <transition id="t2">
    <name>
      <text>Make_roll</text>
    </name>
  </transition>
  <transition id="t3">
    <name>
      <text>Place_cup</text>
    </name>
  </transition>
  <transition id="t4">
    <name>
      <text>Place_roll</text>
    </name>
  </transition>
  <transition id="t5">
    <name>
      <text>Place_embers</text>
    </name>
  </transition>
  <transition id="t6">
    <name>
      <text>Fan_embers</text>
    </name>
  </transition>
  <transition id="t7">
    <name>
      <text>Cool_pit</text>
    </name>
  </transition>
  <transition id="t8">
    <name>
      <text>Collect</text>
    </name>
  </transition>
  <transition id="t9">
    <name>
      <text>Preparations</text>
    </name>
  </transition>
  <transition id="t10">
    <name>
      <text>Clean_soil</text>
    </name>
  </transition>
  <transition id="t11">
    <name>
      <text>Reheat_roll</text>
    </name>
  </transition>
  <transition id="t12">
    <name>
      <text>Store</text>
    </name>
  </transition>
  <transition id="t13">
    <name>
```

```
      <text>Dig_roll_and_cup</text>
    </name>
  </transition>
  <arc id="a0" source="p0" target="t9">
    <inscription>
      <text>1</text>
    </inscription>
  </arc>
  <arc id="a1" source="t9" target="p1">
    <inscription>
      <text>1</text>
    </inscription>
  </arc>
  <arc id="a2" source="t9" target="p3">
    <inscription>
      <text>1</text>
    </inscription>
  </arc>
  <arc id="a3" source="p2" target="t1">
    <inscription>
      <text>1</text>
    </inscription>
  </arc>
  <arc id="a4" source="t1" target="p4">
    <inscription>
      <text>1</text>
    </inscription>
  </arc>
  <arc id="a5" source="p5" target="t3">
    <inscription>
      <text>1</text>
    </inscription>
  </arc>
  <arc id="a6" source="p4" target="t3">
    <inscription>
      <text>1</text>
    </inscription>
  </arc>
  <arc id="a7" source="t0" target="p5">
    <inscription>
      <text>1</text>
    </inscription>
  </arc>
  <arc id="a8" source="t3" target="p6">
    <inscription>
      <text>1</text>
    </inscription>
  </arc>
  <arc id="a9" source="p7" target="t4">
    <inscription>
      <text>1</text>
    </inscription>
  </arc>
  <arc id="a10" source="p6" target="t4">
    <inscription>
      <text>1</text>
    </inscription>
  </arc>
  <arc id="a11" source="t4" target="p8">
    <inscription>
      <text>1</text>
```

```
    </inscription>
  </arc>
  <arc id="a12" source="p8" target="t5">
    <inscription>
      <text>1</text>
    </inscription>
  </arc>
  <arc id="a13" source="t5" target="p9">
    <inscription>
      <text>1</text>
    </inscription>
  </arc>
  <arc id="a14" source="p10" target="t8">
    <inscription>
      <text>1</text>
    </inscription>
  </arc>
  <arc id="a15" source="t8" target="p11">
    <inscription>
      <text>1</text>
    </inscription>
  </arc>
  <arc id="a16" source="p1" target="t10">
    <inscription>
      <text>1</text>
    </inscription>
  </arc>
  <arc id="a17" source="t10" target="p12">
    <inscription>
      <text>1</text>
    </inscription>
  </arc>
  <arc id="a18" source="p12" target="t0">
    <inscription>
      <text>1</text>
    </inscription>
  </arc>
  <arc id="a19" source="p11" target="t11">
    <inscription>
      <text>1</text>
    </inscription>
  </arc>
  <arc id="a20" source="p11" target="t12">
    <inscription>
      <text>1</text>
    </inscription>
  </arc>
  <arc id="a21" source="t12" target="p13">
    <inscription>
      <text>1</text>
    </inscription>
  </arc>
  <arc id="a22" source="t11" target="p10">
    <inscription>
      <text>1</text>
    </inscription>
  </arc>
  <arc id="a23" source="p3" target="t2">
    <inscription>
      <text>1</text>
    </inscription>
```

```
      </arc>
      <arc id="a24" source="t2" target="p7">
        <inscription>
          <text>1</text>
        </inscription>
      </arc>
      <arc id="a25" source="t9" target="p2">
        <inscription>
          <text>1</text>
        </inscription>
      </arc>
      <arc id="a26" source="p9" target="t7">
        <inscription>
          <text>1</text>
        </inscription>
      </arc>
      <arc id="a27" source="p9" target="t6">
        <inscription>
          <text>1</text>
        </inscription>
      </arc>
      <arc id="a28" source="t6" target="p9">
        <inscription>
          <text>1</text>
        </inscription>
      </arc>
      <arc id="a29" source="t7" target="p14">
        <inscription>
          <text>1</text>
        </inscription>
      </arc>
      <arc id="a30" source="p14" target="t13">
        <inscription>
          <text>1</text>
        </inscription>
      </arc>
      <arc id="a31" source="t13" target="p10">
        <inscription>
          <text>1</text>
        </inscription>
      </arc>
    </page>
  </net>
</pnml>
```


C. Raised structure model.


```
<?xml version="1.0" encoding="UTF-8"?>
<pnml xmlns="http://www.pnml.org/version-2009/grammar/pnml">
  <net id="net0" type="http://www.pnml.org/version-2009/grammar/ptnet">
    <name>
      <text>_20210303_raised_structure2_workflow_V2_SF_pn</text>
    </name>
    <page id="page0">
      <name>
        <text>DefaultPage</text>
      </name>
      <place id="p0">
        <name>
          <text>p1</text>
        </name>
```

```
    <initialMarking>
      <text>1</text>
    </initialMarking>
  </place>
  <place id="p1">
    <name>
      <text>p2</text>
    </name>
    <initialMarking>
      <text>0</text>
    </initialMarking>
  </place>
  <place id="p2">
    <name>
      <text>p3</text>
    </name>
    <initialMarking>
      <text>0</text>
    </initialMarking>
  </place>
  <place id="p3">
    <name>
      <text>p4</text>
    </name>
    <initialMarking>
      <text>0</text>
    </initialMarking>
  </place>
  <place id="p4">
    <name>
      <text>p6</text>
    </name>
    <initialMarking>
      <text>0</text>
    </initialMarking>
  </place>
  <place id="p5">
    <name>
      <text>p7</text>
    </name>
    <initialMarking>
      <text>0</text>
    </initialMarking>
  </place>
  <place id="p6">
    <name>
      <text>P8</text>
    </name>
    <initialMarking>
      <text>0</text>
    </initialMarking>
  </place>
  <place id="p7">
    <name>
      <text>p9</text>
    </name>
    <initialMarking>
      <text>0</text>
    </initialMarking>
  </place>
  <place id="p8">
```

```
    <name>
      <text>p5</text>
    </name>
    <initialMarking>
      <text>0</text>
    </initialMarking>
  </place>
  <place id="p9">
    <name>
      <text>p11</text>
    </name>
    <initialMarking>
      <text>0</text>
    </initialMarking>
  </place>
  <place id="p10">
    <name>
      <text>p12</text>
    </name>
    <initialMarking>
      <text>0</text>
    </initialMarking>
  </place>
  <place id="p11">
    <name>
      <text>p13</text>
    </name>
    <initialMarking>
      <text>0</text>
    </initialMarking>
  </place>
  <place id="p12">
    <name>
      <text>p15</text>
    </name>
    <initialMarking>
      <text>0</text>
    </initialMarking>
  </place>
  <place id="p13">
    <name>
      <text>p14</text>
    </name>
    <initialMarking>
      <text>0</text>
    </initialMarking>
  </place>
  <place id="p14">
    <name>
      <text>p22</text>
    </name>
    <initialMarking>
      <text>0</text>
    </initialMarking>
  </place>
  <place id="p15">
    <name>
      <text>p23</text>
    </name>
    <initialMarking>
      <text>0</text>
```

```
    </initialMarking>
  </place>
  <place id="p16">
    <name>
      <text>P10</text>
    </name>
    <initialMarking>
      <text>0</text>
    </initialMarking>
  </place>
  <place id="p17">
    <name>
      <text>p17</text>
    </name>
    <initialMarking>
      <text>0</text>
    </initialMarking>
  </place>
  <place id="p18">
    <name>
      <text>p18</text>
    </name>
    <initialMarking>
      <text>0</text>
    </initialMarking>
  </place>
  <place id="p19">
    <name>
      <text>p16</text>
    </name>
    <initialMarking>
      <text>0</text>
    </initialMarking>
  </place>
  <place id="p20">
    <name>
      <text>p19</text>
    </name>
    <initialMarking>
      <text>0</text>
    </initialMarking>
  </place>
  <place id="p21">
    <name>
      <text>p20</text>
    </name>
    <initialMarking>
      <text>0</text>
    </initialMarking>
  </place>
  <place id="p22">
    <name>
      <text>p21</text>
    </name>
    <initialMarking>
      <text>0</text>
    </initialMarking>
  </place>
  <place id="p23">
    <name>
      <text>p24</text>
```

```
      </name>
      <initialMarking>
        <text>0</text>
      </initialMarking>
    </place>
    <place id="p24">
      <name>
        <text>p25</text>
      </name>
      <initialMarking>
        <text>0</text>
      </initialMarking>
    </place>
    <transition id="t0">
      <name>
        <text>Preparations</text>
      </name>
    </transition>
    <transition id="t1">
      <name>
        <text>Dig_pit</text>
      </name>
    </transition>
    <transition id="t2">
      <name>
        <text>Make_cup</text>
      </name>
    </transition>
    <transition id="t3">
      <name>
        <text>Make_roll</text>
      </name>
    </transition>
    <transition id="t4">
      <name>
        <text>Build_dome</text>
      </name>
    </transition>
    <transition id="t5">
      <name>
        <text>Place_pebbles</text>
      </name>
    </transition>
    <transition id="t6">
      <name>
        <text>Place_firewood</text>
      </name>
    </transition>
    <transition id="t7">
      <name>
        <text>Place_cup</text>
      </name>
    </transition>
    <transition id="t8">
      <name>
        <text>Place_net</text>
      </name>
    </transition>
    <transition id="t9">
      <name>
        <text>Place_roll</text>
```

```
    </name>
  </transition>
  <transition id="t10">
    <name>
      <text>Light_dome</text>
    </name>
  </transition>
  <transition id="t11">
    <name>
      <text>Remove_cup</text>
    </name>
  </transition>
  <transition id="t12">
    <name>
      <text>Add_firewood</text>
    </name>
  </transition>
  <transition id="t13">
    <name>
      <text>Cool_dome</text>
    </name>
  </transition>
  <transition id="t14">
    <name>
      <text>Fix_dome2</text>
    </name>
  </transition>
  <transition id="t15">
    <name>
      <text>Fire_stops</text>
    </name>
  </transition>
  <transition id="t16">
    <name>
      <text>Open_dome</text>
    </name>
  </transition>
  <transition id="t17">
    <name>
      <text>Dome_fumes</text>
    </name>
  </transition>
  <transition id="t18">
    <name>
      <text>Fuming_stops</text>
    </name>
  </transition>
  <transition id="t19">
    <name>
      <text>Close_dome</text>
    </name>
  </transition>
  <transition id="t20">
    <name>
      <text>Remove_dome</text>
    </name>
  </transition>
  <transition id="t21">
    <name>
      <text>Remove_roll</text>
    </name>
```

```
    &lt;/transition&gt;
    &lt;transition id="t22"&gt;
      &lt;name&gt;
        &lt;text&gt;Remove_net_and_pebbles&lt;/text&gt;
      &lt;/name&gt;
    &lt;/transition&gt;
    &lt;transition id="t23"&gt;
      &lt;name&gt;
        &lt;text&gt;Fix_dome&lt;/text&gt;
      &lt;/name&gt;
    &lt;/transition&gt;
    &lt;transition id="t24"&gt;
      &lt;name&gt;
        &lt;text&gt;Collect_tar&lt;/text&gt;
      &lt;/name&gt;
    &lt;/transition&gt;
    &lt;transition id="t25"&gt;
      &lt;name&gt;
        &lt;text&gt;Store&lt;/text&gt;
      &lt;/name&gt;
    &lt;/transition&gt;
    &lt;arc id="a0" source="t0" target="p1"&gt;
      &lt;inscription&gt;
        &lt;text&gt;1&lt;/text&gt;
      &lt;/inscription&gt;
    &lt;/arc&gt;
    &lt;arc id="a1" source="p0" target="t0"&gt;
      &lt;inscription&gt;
        &lt;text&gt;1&lt;/text&gt;
      &lt;/inscription&gt;
    &lt;/arc&gt;
    &lt;arc id="a2" source="p2" target="t2"&gt;
      &lt;inscription&gt;
        &lt;text&gt;1&lt;/text&gt;
      &lt;/inscription&gt;
    &lt;/arc&gt;
    &lt;arc id="a3" source="t0" target="p2"&gt;
      &lt;inscription&gt;
        &lt;text&gt;1&lt;/text&gt;
      &lt;/inscription&gt;
    &lt;/arc&gt;
    &lt;arc id="a4" source="t0" target="p3"&gt;
      &lt;inscription&gt;
        &lt;text&gt;1&lt;/text&gt;
      &lt;/inscription&gt;
    &lt;/arc&gt;
    &lt;arc id="a5" source="p1" target="t3"&gt;
      &lt;inscription&gt;
        &lt;text&gt;1&lt;/text&gt;
      &lt;/inscription&gt;
    &lt;/arc&gt;
    &lt;arc id="a6" source="p3" target="t1"&gt;
      &lt;inscription&gt;
        &lt;text&gt;1&lt;/text&gt;
      &lt;/inscription&gt;
    &lt;/arc&gt;
    &lt;arc id="a7" source="t2" target="p4"&gt;
      &lt;inscription&gt;
        &lt;text&gt;1&lt;/text&gt;
      &lt;/inscription&gt;
    &lt;/arc&gt;
```

```
<arc id="a8" source="t1" target="p5">
  <inscription>
    <text>1</text>
  </inscription>
</arc>
<arc id="a9" source="p5" target="t7">
  <inscription>
    <text>1</text>
  </inscription>
</arc>
<arc id="a10" source="p4" target="t7">
  <inscription>
    <text>1</text>
  </inscription>
</arc>
<arc id="a11" source="t7" target="p6">
  <inscription>
    <text>1</text>
  </inscription>
</arc>
<arc id="a12" source="p6" target="t8">
  <inscription>
    <text>1</text>
  </inscription>
</arc>
<arc id="a13" source="t8" target="p7">
  <inscription>
    <text>1</text>
  </inscription>
</arc>
<arc id="a14" source="p7" target="t5">
  <inscription>
    <text>1</text>
  </inscription>
</arc>
<arc id="a15" source="t3" target="p8">
  <inscription>
    <text>1</text>
  </inscription>
</arc>
<arc id="a16" source="t9" target="p9">
  <inscription>
    <text>1</text>
  </inscription>
</arc>
<arc id="a17" source="p9" target="t4">
  <inscription>
    <text>1</text>
  </inscription>
</arc>
<arc id="a18" source="t4" target="p10">
  <inscription>
    <text>1</text>
  </inscription>
</arc>
<arc id="a19" source="t6" target="p11">
  <inscription>
    <text>1</text>
  </inscription>
</arc>
<arc id="a20" source="p11" target="t10">
```

```
      <inscription>
        <text>1</text>
      </inscription>
    </arc>
    <arc id="a21" source="t10" target="p13">
      <inscription>
        <text>1</text>
      </inscription>
    </arc>
    <arc id="a22" source="p12" target="t13">
      <inscription>
        <text>1</text>
      </inscription>
    </arc>
    <arc id="a23" source="p14" target="t11">
      <inscription>
        <text>1</text>
      </inscription>
    </arc>
    <arc id="a24" source="t11" target="p15">
      <inscription>
        <text>1</text>
      </inscription>
    </arc>
    <arc id="a25" source="t5" target="p16">
      <inscription>
        <text>1</text>
      </inscription>
    </arc>
    <arc id="a26" source="p16" target="t9">
      <inscription>
        <text>1</text>
      </inscription>
    </arc>
    <arc id="a27" source="p8" target="t9">
      <inscription>
        <text>1</text>
      </inscription>
    </arc>
    <arc id="a28" source="p13" target="t12">
      <inscription>
        <text>1</text>
      </inscription>
    </arc>
    <arc id="a29" source="t12" target="p13">
      <inscription>
        <text>1</text>
      </inscription>
    </arc>
    <arc id="a30" source="t14" target="p13">
      <inscription>
        <text>1</text>
      </inscription>
    </arc>
    <arc id="a31" source="p13" target="t14">
      <inscription>
        <text>1</text>
      </inscription>
    </arc>
    <arc id="a32" source="p13" target="t15">
      <inscription>
```

```
      <text>1</text>
    </inscription>
  </arc>
  <arc id="a33" source="t15" target="p12">
    <inscription>
      <text>1</text>
    </inscription>
  </arc>
  <arc id="a34" source="t17" target="p19">
    <inscription>
      <text>1</text>
    </inscription>
  </arc>
  <arc id="a35" source="p19" target="t19">
    <inscription>
      <text>1</text>
    </inscription>
  </arc>
  <arc id="a36" source="t19" target="p12">
    <inscription>
      <text>1</text>
    </inscription>
  </arc>
  <arc id="a37" source="t13" target="p17">
    <inscription>
      <text>1</text>
    </inscription>
  </arc>
  <arc id="a38" source="p17" target="t16">
    <inscription>
      <text>1</text>
    </inscription>
  </arc>
  <arc id="a39" source="t16" target="p18">
    <inscription>
      <text>1</text>
    </inscription>
  </arc>
  <arc id="a40" source="p18" target="t17">
    <inscription>
      <text>1</text>
    </inscription>
  </arc>
  <arc id="a41" source="p18" target="t18">
    <inscription>
      <text>1</text>
    </inscription>
  </arc>
  <arc id="a42" source="t18" target="p20">
    <inscription>
      <text>1</text>
    </inscription>
  </arc>
  <arc id="a43" source="p20" target="t20">
    <inscription>
      <text>1</text>
    </inscription>
  </arc>
  <arc id="a44" source="t20" target="p21">
    <inscription>
      <text>1</text>
```

```
        &lt;/inscription&gt;
      &lt;/arc&gt;
      &lt;arc id="a45" source="p21" target="t21"&gt;
        &lt;inscription&gt;
          &lt;text&gt;1&lt;/text&gt;
        &lt;/inscription&gt;
      &lt;/arc&gt;
      &lt;arc id="a46" source="t21" target="p22"&gt;
        &lt;inscription&gt;
          &lt;text&gt;1&lt;/text&gt;
        &lt;/inscription&gt;
      &lt;/arc&gt;
      &lt;arc id="a47" source="p22" target="t22"&gt;
        &lt;inscription&gt;
          &lt;text&gt;1&lt;/text&gt;
        &lt;/inscription&gt;
      &lt;/arc&gt;
      &lt;arc id="a48" source="t22" target="p14"&gt;
        &lt;inscription&gt;
          &lt;text&gt;1&lt;/text&gt;
        &lt;/inscription&gt;
      &lt;/arc&gt;
      &lt;arc id="a49" source="t23" target="p10"&gt;
        &lt;inscription&gt;
          &lt;text&gt;1&lt;/text&gt;
        &lt;/inscription&gt;
      &lt;/arc&gt;
      &lt;arc id="a50" source="p10" target="t23"&gt;
        &lt;inscription&gt;
          &lt;text&gt;1&lt;/text&gt;
        &lt;/inscription&gt;
      &lt;/arc&gt;
      &lt;arc id="a51" source="p10" target="t6"&gt;
        &lt;inscription&gt;
          &lt;text&gt;1&lt;/text&gt;
        &lt;/inscription&gt;
      &lt;/arc&gt;
      &lt;arc id="a52" source="p15" target="t24"&gt;
        &lt;inscription&gt;
          &lt;text&gt;1&lt;/text&gt;
        &lt;/inscription&gt;
      &lt;/arc&gt;
      &lt;arc id="a53" source="t24" target="p23"&gt;
        &lt;inscription&gt;
          &lt;text&gt;1&lt;/text&gt;
        &lt;/inscription&gt;
      &lt;/arc&gt;
      &lt;arc id="a54" source="p23" target="t25"&gt;
        &lt;inscription&gt;
          &lt;text&gt;1&lt;/text&gt;
        &lt;/inscription&gt;
      &lt;/arc&gt;
      &lt;arc id="a55" source="t25" target="p24"&gt;
        &lt;inscription&gt;
          &lt;text&gt;1&lt;/text&gt;
        &lt;/inscription&gt;
      &lt;/arc&gt;
    &lt;/page&gt;
  &lt;/net&gt;
&lt;/pnml&gt;
```

References

1. M Heiner, M Herajy, F Liu, C Rohr, M Schwarick, Snoopy – a unifying petri net tool in *Application and Theory of Petri Nets*, Lecture Notes in Computer Science, eds. S Haddad, L Pomello. (Springer, Berlin) Vol. 7347, pp. 398–407 (2012).
2. WMP van der Aalst, et al., Prom 4.0: Comprehensive support for real process analysis in *Petri Nets and Other Models of Concurrency – ICATPN 2007*, Lecture notes in computer science, eds. J Kleijn, A Yakovlev. (Springer, Berlin) Vol. 4546, pp. 484–494 (2007).
3. ProM, Prom 6.10 (2010).
